# Longitudinal study on the temporal and micro-spatial distribution of *Galba truncatula* in four farms in Belgium as a base for small-scale risk mapping of *Fasciola hepatica*

**DOI:** 10.1186/s13071-014-0528-0

**Published:** 2014-11-26

**Authors:** Johannes Charlier, Karen Soenen, Els De Roeck, Wouter Hantson, Els Ducheyne, Frieke Van Coillie, Robert De Wulf, Guy Hendrickx, Jozef Vercruysse

**Affiliations:** Department of Virology, Parasitology and Immunology, Faculty of Veterinary Medicine, Ghent University, Salisburylaan 133, 9820 Merelbeke, Belgium; Laboratory of Forest Management and Spatial Information Techniques, Faculty of Bioscience Engineering, Ghent University, Coupure Links 653, 9000 Gent, Belgium; Avia-GIS, Risschotlei 33, 2980 Zoersel, Belgium

**Keywords:** Galba truncatula, Fasciola hepatica, Liver fluke, Species distribution, Small-scale, Risk mapping

## Abstract

**Background:**

The trematode parasite *Fasciola hepatica* causes important economic losses in ruminants worldwide. Current spatial distribution models do not provide sufficient detail to support farm-specific control strategies. A technology to reliably assess the spatial distribution of intermediate host snail habitats on farms would be a major step forward to this respect. The aim of this study was to conduct a longitudinal field survey in Flanders (Belgium) to (i) characterise suitable small water bodies (SWB) for *Galba truncatula* and (ii) describe the population dynamics of *G. truncatula*.

**Methods:**

Four *F. hepatica*-infected farms from two distinct agricultural regions were examined for the abundance of *G. truncatula* from the beginning (April 2012) until the end (November 2012) of the grazing season. Per farm, 12 to 18 SWB were selected for monthly examination, using a 10 m transect analysis. Observations on *G. truncatula* abundance were coupled with meteorological and (micro-)environmental factors and the within-herd prevalence of *F. hepatica* using simple comparison or negative binomial regression models.

**Results:**

A total of 54 examined SWB were classified as a pond, ditch, trench, furrow or moist area. *G. truncatula* abundance was significantly associated with SWB-type, region and total monthly precipitation, but not with monthly temperature. The clear differences in *G. truncatula* abundance between the 2 studied regions did not result in comparable differences in *F. hepatica* prevalence in the cattle. Exploration of the relationship of *G. truncatula* abundance with (micro)-environmental variables revealed a positive association with soil and water pH and the occurrence of *Ranunculus* sp. and a negative association with mowed pastures, water temperature and presence of reed-like plant species.

**Conclusions:**

Farm-level predictions of *G. truncatula* risk and subsequent risk for *F. hepatica* occurrence would require a rainfall, soil type (representing the agricultural region) and SWB layer in a geographic information system. While rainfall and soil type information is easily accessible, the recent advances in very high spatial resolution cameras carried on board of satellites, planes or drones should allow the delineation of SWBs in the future.

## Background

Worldwide, the trematode parasite *Fasciola hepatica* causes important economic losses in cattle due to reduced animal productivity, loss of condemned livers and interference with other diseases. In Western Europe, regional herd-level prevalences between 20 and 80% are often reported and the annual median cost of an infected cow has been estimated to be up to € 300 [1,2].

There is an important spatial component in the epidemiology of fasciolosis because it depends on the presence of an intermediate host snail, which in turn depends on specific climatic and environmental conditions for its development. Although several aquatic snail species have been reported as intermediate hosts for *F. hepatica* in Western Europe [3-5], *Galba truncatula* is considered to be by far the most important.

Several spatial distribution models are available that capture regional differences in *F. hepatica* occurrence [6-8]. However, they do not provide sufficient detail to be used for farm-specific risk assessment, nor support farm-specific control strategies. On the other hand, it has been shown that combining farm management information with knowledge of the presence of *G. truncatula* can accurately predict farm infection status [9]. Management factors can relatively easy be collected on a farm (e.g. through standardized questionnaires), but the judgement of suitable habitats for *G. truncatula* is more difficult because the snail’s distribution is highly variable depending on weather and micro-environmental factors.

Currently, very high-resolution (VHR) remote sensing images, either obtained by satellite or remotely piloted aircraft systems (RPAS), are increasingly used in small-scale risk mapping of vector-borne diseases [10,11]. RPAS can capture landscape features at a spatial resolution up to 0.01-0.2 m [12] and provide a promising tool for *F. hepatica* risk mapping. Nevertheless, developing a standardized method to analyse VHR remote sensing images for creating small-scale risk maps requires more information on preferential habitats and temporal distribution of the intermediate snail host. This information can help to select optimal sensors, image analysis methods and sampling procedures for model validation. Therefore, we carried out a longitudinal field survey in two distinct agricultural regions in Flanders (Belgium) to (i) characterise suitable small water bodies (SWB) for *G. truncatula* and (ii) describe the population dynamics of *G. truncatula*.

## Methods

### Study area and small water bodies

The study was conducted in four dairy cattle farms in Flanders in 2012; two farms in the region of Bruges and two farms in the region of Zoersel. These are two different agricultural regions characterised in Bruges by clay ground and in Zoersel sand/loam soils (Databank Ondergrond Vlaanderen, www.dov.vlaanderen.be).

**Figure 1:**
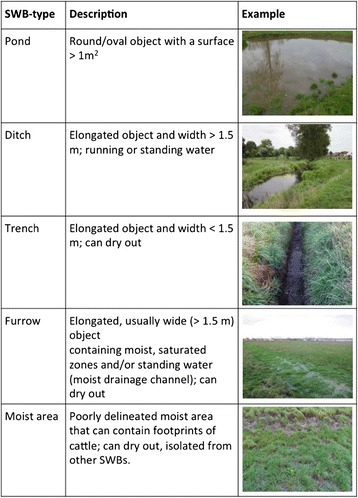
**Definition of five types of small water bodies (SWB) that were identified on the farms and investigated for the presence of** ***Galba truncatula.***

All farms had a liver fluke history based on bulk tank milk ELISA and farm pastures contained permanent and transient potential habitats for *G. truncatula.* These habitats are small water bodies (SWB) and are defined as objects on a grazing pasture that contain temperate or permanent freshwater with a surface >0.5 m^2^. Five different SWB types were classified based on water presence and shape characteristics and literature review [13-15] (Figure 1). Per farm, 12 to 18 SWB were selected for monthly examination (April-November).

### Snail collection

All four farms were examined for freshwater snails from April to November 2012 on a monthly basis from the beginning until the end of the grazing season. A 10 m transect analysis with a search period of 15 min per person [16] was used to sample each habitat. All retrieved snails were morphologically identified according to Devriese *et al*. [17] and Gittenberger *et al*. [18]. The lengths of the *G. truncatula* snails were measured in order to differentiate juvenile (≤4.5 mm) from adult specimens (>4.5 mm) [19]. After the measurement, the snails were released in their natural habitats in order to disturb as little as possible the *G. truncatula* population dynamics.

### Infection status cattle

Faecal and blood samples were taken from 5–8 animals per age category (adult cows, first season grazing calves and, if present, second season grazing calves) in each farm. Samples were taken before grazing season (April 2012), after grazing season (November 2012) and in winter (March 2013) to investigate the concurrent *F. hepatica* infection dynamics in the herd. Faecal samples were assessed by the sedimentation/flotation technique [20] and copro-antigen ELISA (Bio-X Diagnostics, Jemelle, Belgium). Serum samples were assessed for *F. hepatica* antibodies with the SVANOVIR^©^*F. hepatica*-Ab ELISA (Boehringer Ingelheim Svanova, Uppsala, Sweden) according to the manufacturer’s instructions.

### Collection of weather factors and SWB characteristics

Two main components were monitored to describe the abundance pattern of *G. truncatula* in each SWB: weather factors and SWB characteristics.

Weather factors were monitored during the whole study period (April-November). Data of daily minimum, maximum and mean temperature were obtained from the closest meteorological station provided by the Royal Meteorological Institute, Belgium (RMI). These stations were located at a maximal distance of 18 km from the farms monitored. In Bruges, a mobile weather station (Campbell scientific) was set up and monitored precipitation, soil (5 cm in the soil), surface (5–15 cm above the soil) and air temperature (95–105 cm above the soil). The data from the mobile weather station were used to validate the data from the RMI.

SWB characteristics were monitored monthly from August to November using a checklist that included management factors and (micro-)environmental characteristics of the SWB. For management factors, whether SWBs were fenced or mowed at the time of visit was recorded. The (micro-)environmental factors monitored were: water flow (yes/no), trampled soil (yes/no), pH and temperature of soil and water and the occurrence of potential indicator plants: rushes/sedges (i.e. Juncaceae, Cyperaceae), reed-like species (*Phragmites* sp*., Typha* sp.*,)* and buttercups *(Ranunculus* sp.).

### Statistical analysis

First, a negative binomial regression model with robust standard errors was used to assess univariate relationships between snail abundance (dependent variable) and small water body, weather, pasture and (micro-)environmental variables (independent variables). The observed snail counts indicated different SWB preferences according to the region. However, the amount of available data was not sufficient to test the interaction term between SWB-type and region. Next, variables available over the whole study period (April-November) were used in a multivariate model with the same structure as described above. The choice of the negative binomial model was based on explorative work where the relationship with snail abundance was first modelled using a multivariate Poisson model. The χ2 goodness-of-fit test as well as dispersion parameter indicated strong overdispersion in the Poisson model. A negative binomial model fitted the data significantly better, as indicated by the dispersion parameter in this model. A Vuong-test [21] was applied to compare the negative binomial with a zero-inflated negative binomial model. The test was not significant, suggesting the negative binomial model provided an adequate model fit. Finally, a generalized estimating equations (GEE) approach was used to account for repeated observations within search location and provide population-averaged parameter estimates [22]. Standard errors and statistical significance were based on Wald tests. All analyses were carried out with the GENMOD procedure in SAS version 9.3 (SAS Institute Inc., Cary, NC, USA).

## Results

### Snail habitats

**Figure 2:**
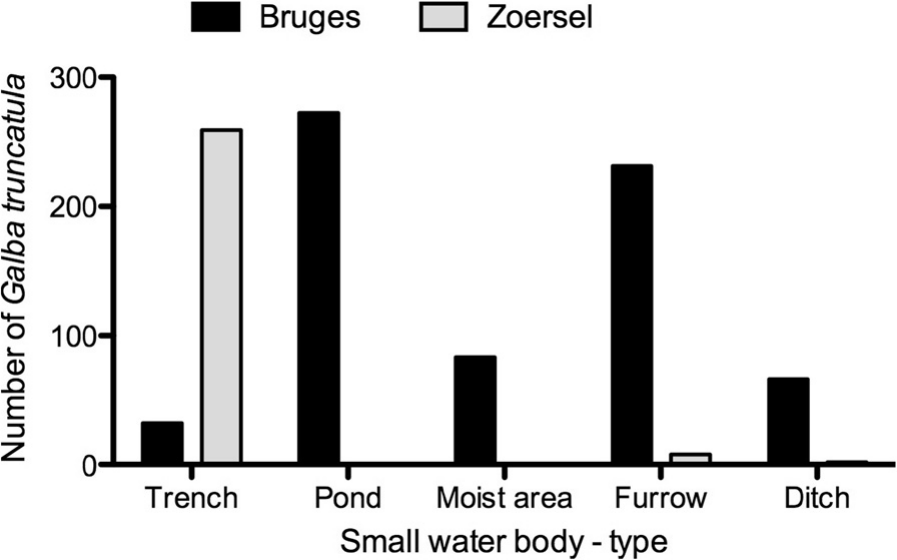
**Observed number of** ***Galba truncatula*** **per small water body-type and region.**

In total, 54 SWB were selected for monthly snail collection and contained 9 ponds, 13 ditches, 11 trenches, 15 furrows and 6 moist areas. There was a difference in distribution of SWB-type between the regions as we searched 31 SWB in Bruges and 23 SWB in Zoersel. The number of collected *G. truncatula* per SWB-type and region is shown in Figure 2.

### Snail sampling

**Figure 3:**
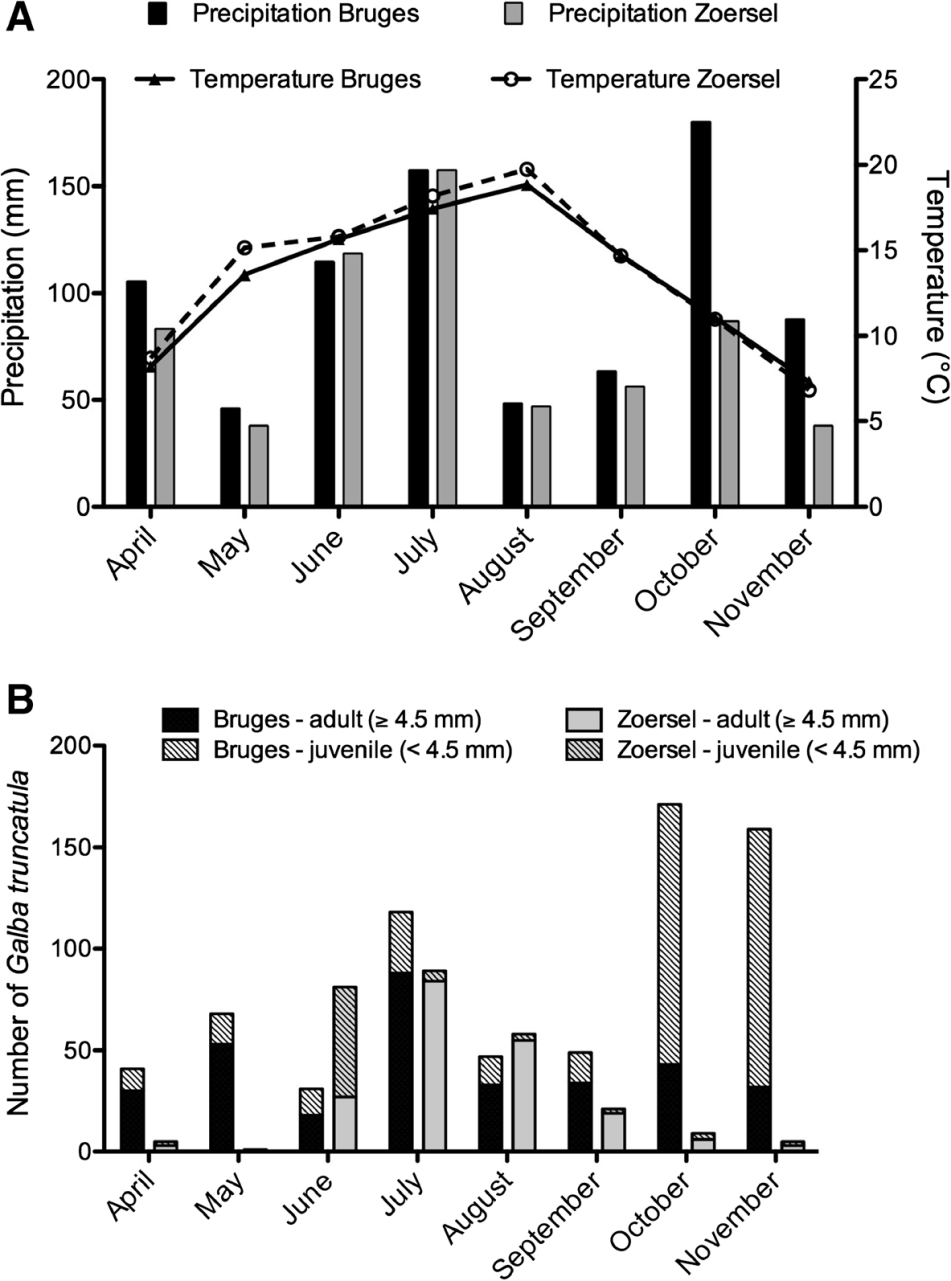
**Mean monthly precipitation and temperature (A) and overall abundance of juvenile and adult** ***G. truncatula*** **snails (B) in the region of Bruges and Zoersel during the study period.**

In total 953 *G. truncatula* snails were found. Other freshwater snails identified were *Planorbis* sp. (*N* = 2309), *Succinea* sp. (*N* = 838), *Lymnaea palustris*, *Lymnaea stagnalis, Radix* sp. and *Bythinia* sp. The abundance and category of *G. truncatula* collected over the study period is shown in Figure 3. A peak of adult *G. truncatula* was observed for both regions in July. In Bruges, a second peak of *G. truncatula*, mostly juveniles, was observed in October/November whereas the population in the region of Zoersel appeared to die off.

### Factors associated with the intermediate host abundance

Temperature and rainfall data obtained from the RMI showed high correlations with the data obtained from the self-placed mobile weather station (Pearson *R* = 0.97 for both monthly temperature and rainfall), indicating that we could rely on RMI data to make inferences on the whole study area and to use in the statistical model. In addition, the average temperature of soil and surface were highly correlated with air temperature (Pearson *R* = 0.97 and 0.98, respectively), suggesting that we could use air temperature solely to explore potential relationships between temperature and snail abundance.

The results of the multivariate negative binomial regression model are given in Table 1. Over the 4 sampled farms, *G. truncatula* was significantly associated with SWB-type, region and total monthly precipitation, but not with monthly temperature. Pairwise comparison indicated that trenches contained significantly more *G. truncatula* than the other SWB-types and ponds contained more *G. truncatula* than ditches, while other comparisons were not significantly different. The region of Bruges showed significantly higher *G. truncatula* abundances than the region of Zoersel. Total monthly rainfall was positively associated with snail abundance. The univariate negative binomial regression models to assess the relationship of *G. truncatula* abundance with management and (micro-)environmental variables showed that abundance of *G. truncatula* was positively associated with soil and water pH and the occurrence of *Ranunculus* sp. and negatively associated with mowing, water temperature and reed-like species. No association was observed with fencing, water flow, trampling, soil temperature and the presences of rushes/sedges (Table 2).

**Table 1:** **Multivariate negative binomial regression model to evaluate the associations between snail abundance and predictor variables monitored throughout the study period**

**Variable**	**Regression coefficient**	**Standard error**	***P*** **-value**
Intercept	−0.532	0.577	0.37
SWB-type			< 0.001
Trench	3.823	0.732	< 0.001
Pond	1.292	0.548	0.019
Moist area	0.831	0.699	0.235
Furrow	0.879	0.509	0.084
Ditch	-	-	
Region			< 0.001
Zoersel	−2.754	0.552	
Bruges	-	-	
Total monthly rainfall (mm)	0.006	0.003	0.028

**Table 2:** **Results from univariate negative binomial regression models to evaluate the associations of** ***G. truncatula*** **abundance with management and (micro-)environmental variables**

**Variable**	**Regression coefficient**	**Standard error**	***P*** **-value**
Fencing			0.320
Yes	−0.592	0.460	
Partial	−0.057	0.217	
No	-	-	
Mowed			0.010
Yes	−0.621	0.240	
No	-	-	
Water flow			0.399
Standing water	−0.299	0.354	
Running water	-	-	
Trampled			0.896
Yes	−0.049	0.379	
No	-	-	
Rushes/sedges			0.572
Yes	0.186	0.330	
No	-	-	
Reed			<0.001
Yes	−0.855	0.253	
No	-	-	
Ranunculus sp.			0.016
Yes	0.616	0.255	
No	-	-	
Water pH	0.399	0.066	<0.001
Soil pH	0.704	0.239	0.003
Water temperature (°C)	−0.074	0.024	0.002
Soil temperature (°C)	−0.005	0.036	0.902

### Infection status of cattle

**Figure 4:**
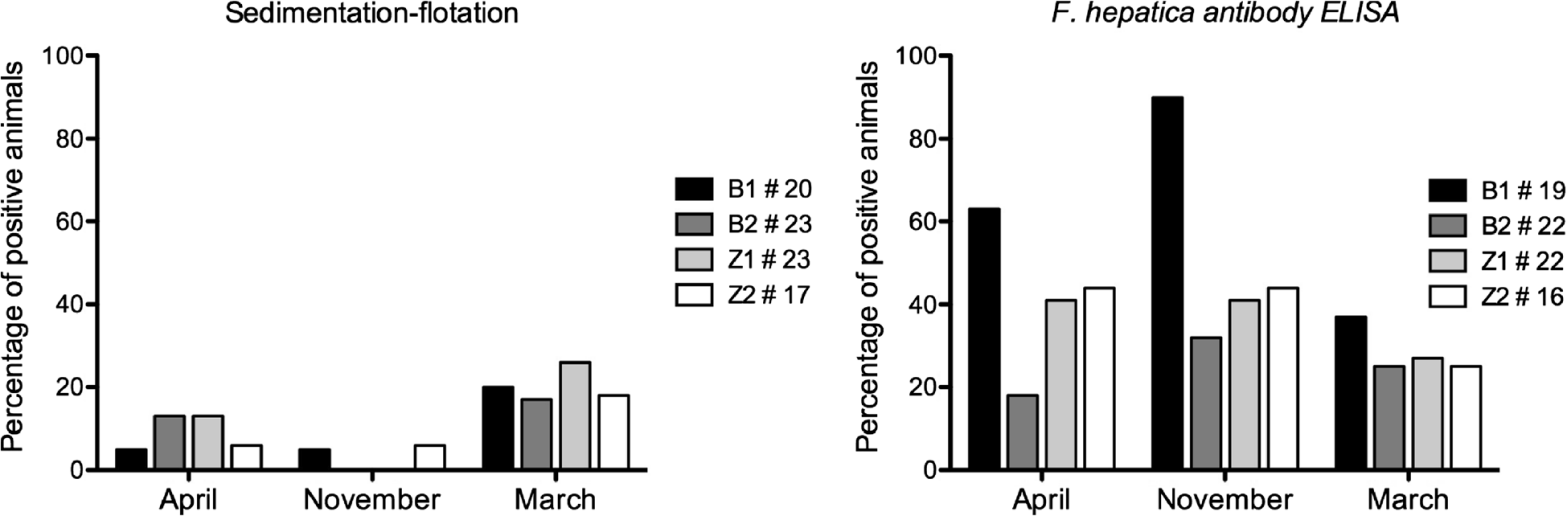
***F. hepatica*** **prevalence in the four investigated farms at three time points by means of two diagnostic methods.**

Between 15 and 23 animals spread over the age categories present at each farm were sampled. The results of the copro-antigen ELISA (results not shown) and sedimentation-flotation method were highly similar. The results of the sedimentation-flotation method and *F. hepatica* antibody ELISA are shown in Figure 4. The average number of animals shedding eggs was remarkably higher in March (21%) than November (3%). The antibody-ELISA showed systematically a higher percentage of positive animals. The highest number of positive cows was observed in November, while in March still 30% of the cows tested positive. No clear difference in prevalence was found between the two regions.

## Discussion

A technology to reliably assess the spatial distribution of intermediate host snail habitats on farms would be a major step forward towards the development of farm-specific risk maps and management of fasciolosis. Our study confirms previous studies that there are important differences in *G. truncatula* abundance between SWB-types [13,14]. Overall, most snails were found in trenches. This may be a surprising result because trenches mostly did not contain water. However, due to their limited width they were often covered by vegetation, staying moisturized underneath. *G. truncatula* were also found in all other types, although to a lesser extent. Nonetheless, the observed differences in *G. truncatula* abundance between SWB-types can be region-specific. First, we observe that despite taking into account SWB-type in our regression model, the region in itself remained an important factor to explain differences in population size and dynamics. Similarly, in another cross-sectional survey in Belgium, Caron et al. [3] found that the geographical location was the best explanatory variable for the presence of *G. truncatula* in ponds, in contrast to type and depth of the pond that were no significant explanatory variables.

Our study failed to assess the importance of reservoir (permanent) habitats vs. extension (temporary) habitats of *G. truncatula* [23]. It is difficult to discriminate between these types of habitats based on a simple definition. Most of the sampled habitats in our study had the possibility of drying out. Because extension habitats need to be connected to reservoir habitats an unambiguous delineation or discrimination of both is often difficult. After a literature review, we used the classification of SWB as described in Figure 1 and maintained that throughout the study.

For the prediction of fasciolosis, it is generally assumed that rainfall is an important determinant of annual risk, whereas temperature in particular determines when pasture becomes dangerous [24]. Our study confirms the effect of rainfall on the magnitude of the snail population, which reached peak levels following high rainfall in a particular month. Considering the effect of temperature, it is known that a temperature above 10°C is essential for egg laying by *G. truncatula* [25]. Recent studies have looked at the effects of temperature on snail growth and the development of eggs, miracidial and intramolluscan stages of *F. hepatica* [26-28] whereas no studies are available on the effects on the population dynamics of *G. truncatula*. Our study suggest that in regions with a temperate climate, once temperature is higher than 10°C, rainfall is the main driver of *G. truncatula* abundance and temperature has no large influence.

There was a clear difference in the number of generations of *G. truncatula* in the 2 studied regions. In Zoersel, there was only 1 clear generation over the study period with a peak in abundance in July. This suggests that ‘summer infection’ [29] is likely the main transmission pattern in this region. Summer infection was also the predominant transmission pattern in another study recently conducted in Sweden [30]. In Bruges, there were 2 clear peaks in abundance: one in July and one in October. Whereas the first peak can result in the ‘summer infection” transmission pattern, the second peak consisted mainly of juvenile snails. This generation could form the basis of an important overwintering snail population and suggests ‘winter infection’ [29] could potentially play a more important role in transmission than previously thought in this region.

Exploration of the relationship of *G. truncatula* abundance with (micro-)environmental variables revealed interesting associations. First, we found a positive association with soil and water pH. This contrasts with often retrieved statements that *G. truncatula* prefers mildly acidic soils [31]. Nonetheless, positive associations between *G. truncatula* and soil pH have been reported previously [3,32]. The association with plant species contributes to knowledge of the use of indicator plants to evaluate suitability of habitats for *G. truncatula* [13] while the negative association with mowed pastures may suggest that mowing could be used as a pasture sanitation strategy to reduce *F. hepatica* risk [6].

The clear differences in *G. truncatula* abundance between the 2 regions studied did not result in comparable differences in *F. hepatica* prevalence in the cattle. Although the herd prevalence of *F. hepatica* is lower in Zoersel than Bruges [33], the within-herd prevalence of the studied farms was not. This could be caused by the fact that, in contrast to Zoersel, the cattle in Bruges had received annual flukicide treatment for several years and this may thus have reduced the impact of the environmental risk. On the other hand, in Zoersel we found other snail species such as *Succinea* sp. in a higher relative proportion, and other snail species naturally infected with *F. hepatica* have been recently documented [4,5,34,35]. It has been suggested these species may play an important role in transmission when *G. truncatula* is not abundant [36].

Our study suggests that farm-level predictions of *G. truncatula* risk and subsequent risk for *F. hepatica* occurrence would require several layers in a geographic information system (GIS). Besides a layer with rainfall and soil type (representing the agricultural region), a layer with SWB localisation will be needed. This SWB layer forms the bottleneck of the map and new classification methodologies are needed to identify these small water bodies, which mostly cannot be found on regular hydrology maps. The recent advances in the very high spatial resolution cameras carried on board of satellites, planes or drones allow the delineation of the SWBs [37]. Images with a spatial resolution at cm level are now readily available and can be used for a rapid assessment on the pastures. Drones provide the advantage that they can be used at any time required, in contrast to satellites that have to be programmed beforehand. Moreover images resulting from the satellite may be influenced by cloud contamination. In terms of spectral resolution, standard Red Green Blue (RGB) camera’s provide sufficient spectral information to create a baseline map of the presence of the SWBs at farm level, however, investigations should determine whether for example cameras with near-infrared channels can provide added value that can be used to delineate the SWBs. The raw images could then be translated into SWBs, using either pixel-based classification methods or segmentation algorithms [38].

This last step could also provide new definitions of SWB and thus avoid uncertainties related to the terminology used [39]. Indeed, the current studies investigating *G. truncatula* habitats use different definitions [13,14], greatly hampering comparison of study results.

## Conclusions

Farm-level predictions of *G. truncatula* risk and subsequent risk for *F. hepatica* occurrence would require a rainfall, soil type (representing the agricultural region) and SWB layer in a geographic information system. While rainfall and soil type information is easily accessible, the recent advances in very high spatial resolution cameras carried on board of satellites, planes or drones should allow the delineation of SWBs in the future.
